# Einsatz des REBOA-Katheters bei unstillbarer oberer gastrointestinaler Blutung mit hämorrhagischem Schock

**DOI:** 10.1007/s00101-023-01278-0

**Published:** 2023-03-29

**Authors:** Richard Steffen, Jürgen Knapp, Matthias Hänggi, Manuela Iten

**Affiliations:** 1 Universitätsklinik für Intensivmedizin, Inselspital, Universitätsspital Bern, Universität Bern, Bern, Schweiz; 2 Klinik für Anästhesiologie und Schmerztherapie, Inselspital, Universitätsspital Bern, Universität Bern, 3010 Bern, Schweiz; 3 Schweizerische Rettungsflugwacht, Rega, Zürich, Schweiz

## Einführung

Der Einsatz der notfallmäßigen endovaskulären Ballonokklusion der Aorta („resuscitative endovascular balloon occlusion of the aorta“, REBOA) wird in den letzten Jahren wieder zunehmend für die Anwendung bei Traumapatienten mit nichtkomprimierbaren Blutungen diskutiert. Hierbei stellen insbesondere Beckenfrakturen und traumatische intraabdominale Blutungen die wichtigsten Indikationen dar [1]. Bislang ist der Einsatz dieses Katheters noch keineswegs als Routinemaßnahme etabliert, sondern vielmehr abhängig von individuellen Therapieentscheidungen einzelner Ärzte. Der REBOA-Katheter wird über eine Schleuse in die A. femoralis communis eingeführt und proximal der entsprechenden Läsion in der Aorta platziert. Durch die Befüllung des Ballons wird eine (partielle) Okklusion der Aorta erreicht, mit folglich einer kompletten oder teilweisen Verminderung des Blutflusses distal des Ballons und somit einer massiven Reduktion des Blutverlustes und meist deutlicher hämodynamischer Stabilisierung des Patienten. Über prähospitale Anwendungen bei Traumapatienten [2] und den Einsatz bei der kardiopulmonalen Reanimation [3] liegen bisher lediglich kleine Pilotstudien oder Fallberichte vor. Zur Anwendung der REBOA bei massiven oberen gastrointestinalen Blutungen finden sich bei einer PubMed-Recherche auch lediglich eine Fallserie mit 8 Patienten aus dem Jahre 2016 [4], 2 Fallberichte von aufgrund einer oberen gastrointestinalen Blutung reanimationspflichtigen Patienten [5, 6] sowie ein Bericht über die Anwendung eines REBOA-Katheters bei einem Patienten mit einer aortoösophagealen Fistel nach onkologischer Lobeketomie [13]. Aufgrund des Charakters als „Ultima-Ratio“-Therapie fehlt eine wissenschaftliche Evaluation ihrer Effizienz – wie beim Traumapatienten – bisher auch hier. Die Anzahl der Patienten, die für eine REBOA überhaupt infrage kommen, lässt sich bis jetzt nur schwer abschätzen. In einer aktuellen retrospektiven Querschnittstudie aus Norwegen (*n* = 804 Patienten) mit massiven Blutungen, wurde in 6,6 % aller Fälle ein potenzieller Nutzen einer REBOA-Einlage postuliert; hierbei lag bei 6 von 44 Patienten aus der nichttraumatischen Gruppe eine gastrointestinale Blutung vor [7]. In diesem Fallbericht wird der Einsatz der REBOA bei einem Patienten mit unkontrollierbarer, schwerer oberer gastrointestinaler Blutung vorgestellt und der Nutzen kritisch diskutiert.

## Fallbeschreibung

Der 65-jährige Patient stellte sich bei akuter kardialer Dekompensation im Rahmen einer schweren, progredienten Mitralklappeninsuffizienz vor. Ätiologisch zeigte sich führend eine ischämische Kardiomyopathie mit Zustand nach Myokardinfarkt und Stent-Einlage vor 5 Monaten. Nebenbefundlich bestanden ein tachykardes Vorhofflimmern unter oraler Antikoagulation mit Apixaban sowie eine Hämochromatose und eine subklinische Hypothyreose. Nach kardialer Rekompensation und Bridging der oralen Antikoagulation mit Heparin in therapeutischer Dosierung erging die Indikation zur operativen Mitral- und Trikuspidalklappenrekonstruktion. Der unmittelbar perioperative Verlauf gestaltete sich komplikationslos. Die Plättchenaggregationshemmung mit Clopidogrel wurde perioperativ weitergeführt. Am dritten postoperativen Tag wurde mit Phenprocoumon begonnen, worunter es zu einem Anstieg der International Normalized Ratio (INR) bis maximal 2,57 kam. Am selben Abend trat eine akute – hämodynamisch relevante und transfusionsbedürftige – obere gastrointestinale Blutung auf. In der Gastroskopie fand sich ein ausgedehntes Ulkus ohne aktive Blutungszeichen im Bereich des Bulbus duodeni. Nach Substitution von 5 Erythrozytenkonzentraten, Gerinnungsfaktoren IX, II, VII und X (Beriplex®) sowie Gaben von Vitamin K und Tranexamsäure stabilisierte sich der Patient hämodynamisch. Die Heparingabe wurde im Anschluss in lediglich prophylaktischer Dosierung weitergeführt.

Zwei Tage später kam es zu einer erneuten Episode mit reichlich Meläna und Hämatemesis sowie progredienter Hypotension, sodass von einer neuerlichen massiven gastrointestinalen Blutung ausgegangen werden musste. Der Patient wurde sofort notfallmäßig auf die Intensivstation übernommen. Laborchemisch zeigte sich zum Zeitpunkt der Übernahme eine Hämoglobinkonzentration von 81 g/l bei numerisch normaler Gerinnung (INR 1,0, Fibrinogen 3,2 g/l). In der Blutgasanalyse fiel bereits zu diesem Zeitpunkt eine respiratorisch kompensierte, metabolische Acidose (pH 7,35, Base Excess −5,9 mmol/l, Lactat 5,2 mmol/l) auf. Eine invasive Blutdruckmessung wurde installiert, unmittelbar gefolgt von einer Narkoseeinleitung zur Schutzintubation und der parallelen Anlage eines großlumigen (9 F) Zugangs in die rechte V. femoralis sowie zusätzlich eines dreilumigen zentralen Venenkatheters in die rechte V. jugularis interna. Es entwickelte sich trotz aggressiver Transfusionstherapie ein schwerer hämorrhagischer Schock, sodass 45 min nach dem Beginn der Versorgung bei nun drohendem Kreislaufstillstand der Entscheid zur notfallmäßigen Einlage einer REBOA in die Zone I (Platzierung des Ballons zwischen dem Abgang der linken A. subclavia und dem Truncus coeliacus) fiel. Hierzu wurde eine 7‑F-Schleuse ultraschallgesteuert in die rechte A. femoralis eingelegt und anschließend ein ER-REBOA™-PLUS-Catheter (Prytime Medical™, Boerne, TX, USA) auf 46 cm ab Schleusenende eingeführt [1, 8]. Die Zeitdauer vom Entscheid zur REBOA bis zur erfolgreichen Aortenokklusion betrug 17 min.

**Figure 1:**
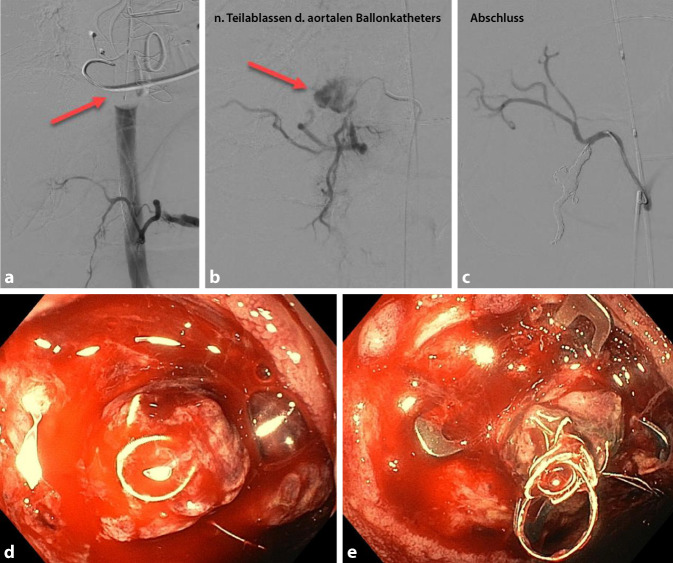

**Figure 2:**
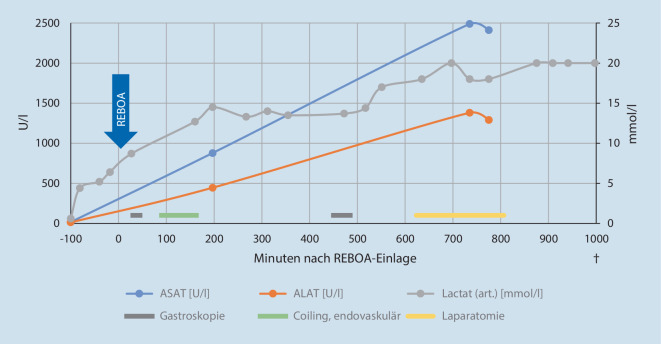

Unmittelbar mit Okklusion der Aorta konnte – unter paralleler Transfusion von Blutprodukten – eine hämodynamische Stabilisierung erreicht werden. In der sofort folgenden Gastroskopie konnten nun Hinweise auf eine arterielle Ulkusblutung – mutmaßlich aus dem Versorgungsgebiet der A. gastroduodenalis – dargestellt werden, bei mehrfachem probatorischem Ablassen des Ballons während der Gastroskopie zeigten sich jeweils sofort wieder eine massive Hämorrhagie und ein Sistieren bei erneutem Füllen des Ballons. Endoskopisch gelang es leider nicht, die Blutung zu stoppen, sodass notfallmäßig endovaskulär die Aa. supraduodenalis und gastroduodenalis gecoilt werden mussten (Abb. 1a–c). Während der Embolisation wurde der Ballon des REBOA-Katheters intermittierend kurzzeitig abgelassen, und unter radiologischer Kontrolle konnte nach der Intervention auch bei entlastetem Ballon schließlich ein Sistieren der Blutung nachgewiesen werden. Der Patient stabilisierte sich zunächst klinisch und laborchemisch; die Hämoglobinkonzentration zeigte einen adäquaten Anstieg und hielt sich über 5 h ohne weitere Transfusionen stabil um 105 g/l. Am Abend desselben Tages kam es jedoch zu einer erneuten massiven Blutung mit hämodynamischer Schocksymptomatik sowie erneutem Abfall der Hämoglobinkonzentration auf minimal 61 g/l trotz forcierter Transfusion. Wir hatten die Schleuse und den REBOA-Katheter sicherheitshalber in situ belassen und konnte so verzugslos erneut die Aorta okkludieren. In der notfallmäßigen Regastroskopie fand sich (bei intermittierend abgelassenem Ballon) wieder eine aktive Blutung aus dem vorbekannten Ulkus. Als Notfalltherapie wurde ein sog. Over-the-scope clip (OTSC) appliziert (Abb. 1d, e). Die Blutung konnte jedoch dadurch nicht suffizient kontrolliert werden, sodass bei fehlenden endoskopischen und endovaskulären Therapieoptionen der interdisziplinäre Entscheid zur offenen, partiellen Duodenektomie und Ligatur der A. gastroduodenalis erging. Der Patient wurde mit geblocktem Ballon in den OP verbracht. Da absehbar war, dass die Blutung nicht innerhalb der empfohlenen maximalen Aortenokklusionszeit von 45 min kontrolliert sein würde, entschieden wir uns, den Ballon während des operativen Eingriffs intermittierend zu entblocken, um eine Ischämie der abdominellen Organe zu vermeiden. Im offenen Situs während der Laparotomie konnte die fehlende Perfusion der A. hepatica propria bei aktivierter REBOA nachgewiesen werden, wobei sich nach Ablassen der Okklusion jeweils eine suffiziente Pulsation zeigte. Die Leber zeigte sich während des operativen Eingriffs etwas abgerundet, jedoch vital wirkend. Der Magen imponierte dunkel verfärbt, aber ebenfalls vital und das Colon transversum nur mäßig dilatiert. Klinisch präsentierte der Patient in den Phasen mit ungeblocktem Ballon eine massive Vasoplegie, sodass trotz aggressiver Volumensubstitution sehr hohe Katecholamindosierungen zur Aufrechterhaltung eines minimalen systemischen Blutdruckes nötig waren (Maximaldosierung: Adrenalin 0,2 µg/kgKG und min, Noradrenalin 0,4 µg/kgKG und min, Vasopressin 0,067 U/min). Auch nach Gabe von Hydrokortison konnten die Dosierungen nicht relevant reduziert werden. Differenzialdiagnostisch wurde auch an eine septische Ursache bei wiederholten Interventionen gedacht, sodass eine empirische antibiotische Therapie mit Piperacillin/Tazobactam und Vancomycin initialisiert wurde. Laborchemisch zeigte sich schließlich ein massiver Anstieg der Lactatkonzentration (bis maximal 20 mmol/l) sowie der Transaminasen (ASAT bis 2500 U/l, ALAT bis 1500 U/l) im Serum (Abb. 2). Zudem aggravierte in diesem Zeitraum die metabolische Acidose mit einem Base Excess bis −7 mmol/l, und es trat eine Hyperkaliämie bis maximal 6,6 mmol/l auf. Leider konnte im weiteren Verlauf trotz fortgesetzter Massivtransfusion (insgesamt 29 Erythrozytenkonzentrate, 24 Plasmaeinheiten, 8 Thrombozytenkonzentrate) und kontinuierlicher Korrektur der Gerinnungssituation (18 g Fibrinogen, 3000 IE Beriplex®, 6 mg Faktor VII) keine hämodynamische Stabilisation unter entblocktem Ballon der REBOA erreicht werden. Der Patient verstarb gut 3 h nach Abschluss des operativen Eingriffs unter laufender Therapie bei progredientem Multiorganversagen auf der Intensivstation.

## Diskussion

Wir konnten mit diesem Fall die Durchführbarkeit der aortalen Ballonokklusion in Zone I und primäre hämodynamische Stabilisierung bei einem Patienten mit schwerster oberer gastrointestinaler Blutung – im Sinne einer überbrückenden Notfallmaßnahme – aufzeigen. Nach Einlage und Blocken des Ballons kam es jeweils zu einem relevanten Anstieg der invasiv gemessenen Blutdruckwerte mit deutlicher Zunahme der Blutdruckamplitude. Zudem konnte unter endoskopischer Sicht bei gefülltem Ballon jeweils ein unmittelbares Sistieren der Blutung beobachtet werden. Auch am offenen Situs während des operativen Eingriffs konnte die Effektivität der REBOA zur Blutungskontrolle bei oberer gastrointestinaler Blutung bestätigt werden. Dennoch verstarb der Patient leider trotz maximaler Therapie im Multiorganversagen. Als beitragend zu diesem Verlauf müssen die Phasen des hämorrhagischen Schocks mit globaler Minderperfusion, die rezidivierenden Perfusionsunterbrechungen der abdominalen Organe durch den Einsatz der REBOA und insbesondere in Bezug auf die Leber die bestehende Hämochromatose genannt werden. Der resultierende Circulus vitiosus aus Blutung, Kreislaufschock und konsekutivem Gerinnungsversagen war letztlich nicht zu beherrschen. Die im Verlauf – insbesondere während des operativen Eingriffs – dann fehlende Stabilisierbarkeit des Patienten unter entblocktem Ballon interpretierten wir als Resultat einer schweren systemischen Entzündungsreaktion mit Reperfusionsschaden nach mehreren hämorrhagischen Schockereignissen und insgesamt 4 (letztlich einer offen operativen) Interventionen im Anschluss an einen großen herzchirurgischen Eingriff. Der genaue Pathomechanismus dieser Reperfusionsreaktion mit massiver Vasoplegie ist bisher nicht vollständig geklärt, scheint aber multifaktoriell zu sein und mehrere Mechanismen zu beinhalten. Eine zentrale Rolle dürften hierbei freie Sauerstoffradikale einnehmen, welche im Rahmen der Lipidperoxidation während der Gewebsischämie anfallen und die Zellmembranen direkt schädigen. Dieser Effekt wird zudem durch die Freisetzung von entzündungsfördernden Mediatoren verstärkt, welche dann Entzündungsreaktionen in entfernten Organen auslösen und dadurch ein Multiorganversagen aggravieren können. Die wichtigste und effektivste Strategie zur Vermeidung dieser Reperfusionsreaktion besteht darin, die Ischämiezeit zu verkürzen. Dies kann bei Anwendung der REBOA in erster Linie durch Anstreben einer frühzeitigen partiellen oder intermittierenden Okklusion erreicht werden. Weitere Therapiemöglichkeiten wie eine therapeutische Hypothermie oder die Gabe von Antioxidanzien konnten bisher keinen Vorteil für den Patienten zeigen [14].

Kritisch muss in dem dargestellten Fall die gewisse Verzögerung bis zum Entscheid zur offenen, partiellen Duodenektomie nach der Rezidivblutung diskutiert werden. Hier hätte eine frühzeitige Involvierung der Viszeralchirurgie die Entscheidungswege möglicherweise verkürzt. Bei Patienten mit hämorrhagischem Schock aufgrund einer oberen gastrointestinalen Blutung sollten daher unserer Ansicht nach immer die Gastroenterologie/Endoskopie, Angiologie/interventionelle Radiologie und Viszeralchirurgie gleichzeitig involviert werden und ein gemeinsamer, klar definierter und abgestufter Maßnahmenplan erstellt werden, sodass die therapeutischen Maßnahmen bei Misslingen einer endoskopischen bzw. endovaskulären Blutungskontrolle ohne Zeitverzug eskaliert werden können. Es bleibt unklar, ob ein frühzeitigerer Entscheid zum Einsatz der REBOA in diesem Fall sinnvoll gewesen wäre. Einige Autoren erachten aber eine präventive Einlage, ohne primäre Okklusion, bei hämodynamisch instabilen Patienten mit hohem Schockindex und/oder Lactat im Serum als gerechtfertigt, sodass jederzeit die Option besteht, eine Ballonokklusion vorzunehmen, falls sich die klinische Situation des Patienten verschlechtert [12].

Während eine Notfalltherapie mittels REBOA inzwischen bei traumatischen und postpartalen Hämorrhagien regelmäßig in Betracht gezogen und angewendet wird, scheint bisher die Indikation bei einer hämodynamisch relevanten gastrointestinalen Blutung allerdings noch selten gestellt zu werden. So wurden beispielsweise selbst an einer Klinik der Maximalversorgung der University of Maryland, Baltimore, gerade einmal 3 Anwendungen innerhalb von 3 Jahren bei dieser Indikation beschrieben [9]. In dieser Studie betrug die Zeit bis zur Einlage der REBOA nach Detektion einer Hypotonie oder einer klinischen Blutung zwischen 62 und 210 min und erfolgte damit (ähnlich wie in unserem Fall) erst bei fortgeschrittenem Schockgeschehen. Weiter wurde eine Anwendung in Deutschland bei einer gastrointestinalen Blutung bei aortoösophagealer Fistel beschrieben [13].

Nutzen und Risiken der REBOA müssen – insbesondere im Hinblick auf die fehlende Evidenz – in jedem Fall kritisch gegeneinander abgewogen werden. Hierbei treten verfahrensbedingte Komplikationen wie Bildung von Pseudoaneurysmata, Gefäßverletzungen sowie Fehllagen des Katheters mittlerweile durch verbessertes Material sehr selten auf [1, 8]. Es wurde gezeigt, dass die ultraschallgesteuerte Gefäßpunktion in Notfallsituation sicher und effizient durchgeführt werden kann [10]. Gegenüber einer Punktion ohne Sonographie konnte eine höhere Erfolgsrate erreicht werden, und die mechanischen Komplikationen konnten reduziert werden [11]. Demgegenüber sollten aber insbesondere bei einer REBOA in Zone I den systemischen Komplikationen große Aufmerksamkeit geschenkt werden. Diese sind größtenteils auf die verfahrensbezogene vorübergehende Ischämie und anschließende Reperfusion zurückzuführen. Bei Einlage der REBOA in die Zone I kommt es entsprechend zu einer passageren Minderperfusion der Bauchorgane mit möglicher Schädigung von Nieren, Leber, gastrointestinalen Organen oder auch des Rückenmarks. Diese systemischen Auswirkungen stehen hierbei wohl in direktem Zusammenhang mit der totalen Okklusionszeit. Verbindliche Richtlinien zur maximalen Ischämiezeit liegen bis anhin nicht vor, jedoch muss in jedem Fall eine schnellstmögliche definitive Versorgung der Blutung angestrebt werden, und eine Okklusionszeit von mehr als 45 min in der Zone I sollte, wenn immer möglich, vermieden werden [8]. Distale Embolisierungen mit konsekutiven Extremitätenischämien wurden bisweilen als seltene Komplikationen beschrieben. Weiter können möglicherweise aufgrund der abrupt steigenden Nachlast auch kardiopulmonale Komplikationen (Lungenödem, linksventrikuläre Dekompensation) auftreten. Bei einer Anlage der REBOA in Zone III (z. B. im Rahmen von Beckenfrakturen) sind systemische Komplikationen deutlich seltener und können längere Ischämiezeiten (bis 90 min) toleriert werden [1]. Durch die erfolgreiche hämodynamische Verbesserung des Patienten mit nun scheinbar stabiler Situation steigt die Gefahr, dass die weitere Behandlung zu wenig rasch und aggressiv erfolgt, insbesondere, wenn das Behandlungsteam nur wenig Erfahrung mit der REBOA hat. Daher müssen alle in die Behandlung involvierten Disziplinen (Intensivmedizin, Notfallmedizin, Anästhesie, Gastroenterologie, Angiologie, Viszeralchirurgie und weitere) für die Möglichkeiten und insbesondere auch die Limitationen und Gefahren dieser Therapie sensibilisiert werden. Die aortale Ballonokklusion sollte zwingend als Überbrückungsstrategie, welche die definitive Blutungsstillung nicht verzögern darf, gesehen werden, aber andererseits auch möglichst frühzeitig eingesetzt werden. So rasch wie möglich sollte eine partielle oder eine intermittierende Okklusion angestrebt werden, damit eine Organischämie reduziert und die kumulative Okklusionszeit verkürzt werden kann.

Zusammenfassend erscheint der Einsatz der REBOA bei oberer gastrointestinaler Blutung mit hämorrhagischem Schock als Notfalltherapie durchführbar. Eine temporäre, hämodynamische Stabilisierung kann hierdurch erreicht werden, wobei jedoch in jedem Fall eine unverzügliche, permanente Blutungskontrolle angestrebt werden muss. Eine sinnvolle Indikation für die REBOA bei gastrointestinalen Blutungen könnte somit der hämorrhagische Schock sein, wenn beispielsweise außerhalb von Zentrumsspitälern eine sofortige blutungsstillende Intervention (Operation oder Embolisation) nicht möglich ist. Dabei ist allerdings zu beachten, dass das entsprechende Personal adäquat in der Handhabung des Katheters geschult ist.

## Fazit

- Durch die Anlage einer REBOA in Zone I können Blutungen des Körperstamms wie z. B. schwere obere gastrointestinale Blutungen rasch kontrolliert werden.
- Die REBOA bietet somit die Möglichkeit, Patienten mit schwersten gastrointestinalen Blutungen im Rahmen einer Akutbehandlung bis zur definitiven Versorgung durch eine Intervention oder Operation temporär zu stabilisieren.
- Spätestens mit der Entscheidung zur Anlage einer REBOA sollten alle möglicherweise kausal therapeutisch erforderlichen Disziplinen umgehend involviert werden, um eine definitive Blutungskontrolle innerhalb einer möglichst kurzen Okklusionszeit erreichen zu können.
- Obschon der optimale Anlagezeitpunkt der REBOA bislang unbekannt ist, erscheint es, um ein prolongiertes Schockgeschehen zu vermeiden, sinnvoll, diese eher frühzeitig und ggf. präemptiv (d. h. nur Schleusen- und Katheteranlage, ohne Okklusion) einzulegen.
- Die REBOA stellt eine überbrückende Therapie bis zur definitiven Blutungsversorgung dar und darf diese nicht verzögern. Die weiterbehandelnden Disziplinen müssen für die Möglichkeiten und insbesondere auch die Limitationen und Gefahren der REBOA sensibilisiert werden.
